# 

**Published:** 1885-01

**Authors:** 


					﻿io cts. a copy.
JAN.1885.
$1.00 per Y^ar..
i.iai
eJoWALJ
HEALTH
ESTABLISHED 1854
Vi-3 2
0^2.- 1
Office, 75 & 77 Barclay Street, New York
Entered a* Baoond-claaa Mattar at tha Pa»t-O®ca, Xaw Tort.
				

